# Usual Intake of Micronutrients and Prevalence of Inadequate Intake among Chinese Adults: Data from CNHS 2015–2017

**DOI:** 10.3390/nu14224714

**Published:** 2022-11-08

**Authors:** Kun Huang, Hongyun Fang, Dongmei Yu, Qiya Guo, Xiaoli Xu, Lahong Ju, Shuya Cai, Yuxiang Yang, Xiaoqi Wei, Liyun Zhao

**Affiliations:** NHC Key Laboratory of Trace Element Nutrition, National Institute for Nutrition and Health, Chinese Center for Disease Control and Prevention, Beijing 100050, China

**Keywords:** micronutrients, usual intake, prevalence of inadequate, NCI method

## Abstract

Previous studies have used the traditional average-value method to calculate the usual dietary intake of a population, but the results may be biased due to the measurement errors. The aim of this study was to provide an assessment of the usual micronutrient intake and estimate the prevalence of inadequate intake among Chinese adults. Data from the Chinese Nutrition and Health Surveillance 2015–2017 as well as a total of 72,231 subjects aged 18 years and older were included in the analysis. The 24 h recall method combined with the condiment weighing method were used for three consecutive days to collect daily food and condiments intake. The daily intake of 16 micronutrients was calculated based on the Chinese Food Component Tables. The National Cancer Institute (NCI) method was used to estimate the usual intake of micronutrients, and the prevalence of inadequate intake was estimated using the estimated average requirement (EAR) cut-point method. The results showed that, except for sodium, copper, iron (only for males), vitamin E, and phosphorus, the usual intake of micronutrients in Chinese adults was low, and the prevalence of inadequate intake ranged from 38.67 to 97.63%. The prevalence of inadequate calcium and riboflavin intake was more than 90%, and the proportion of individuals with a usual intake of thiamine, vitamin A, potassium, and selenium below EAR also reached 80%. Manganese, magnesium, vitamin C, and zinc were potentially deficient micronutrients, with the prevalence of inadequate intake ranging from 38.67% to 77.09%. However, usual sodium intake was extremely high with an average of 5139.61 mg/day, and only a quarter of Chinese adults were below the World Health Organization (WHO) recommended value. For most micronutrients, the usual dietary intake declined with age and the prevalence of inadequate intake increased with age. Except for zinc, vitamin A, and B-vitamins, the prevalence of micronutrient deficiencies was higher in females than in males in the same age group (*p* < 0.05). Therefore, Chinese adults do not receive enough micronutrients. Effective nutrition supplementary strategies and measures are needed to address these problems.

## 1. Introduction

Suboptimal diet has been a key risk factor for chronic non-communicable diseases for decades. In recent years, the global burden of death and disease due to dietary factors has also attracted widespread attention. In 2017, 11 million deaths and 255 million disability-adjusted life years were attributable to dietary risk factors globally [1]. Additionally, dietary risks factors have been identified as one of the top three causes for global attributable deaths among males and females [2].

Micronutrients, including minerals and vitamins, are mainly derived from food, and play important functions in promoting body growth and maintaining related health [3]. Micronutrients deficiency can lead to several health-threatening outcomes [4,5], for example, a weaker immune system, anemia, neurodegenerative diseases, cardiovascular diseases, cancer, and increased morbidity and mortality [6,7,8,9,10,11]. It is estimated that about 2 billion people worldwide have micronutrient deficiency, mainly in developing countries and regions [12]. In addition, micronutrient deficiencies are expected to result in a loss of about 0.8% to 2.5% of gross domestic product in some developing countries. Therefore, knowledge about the population distribution of micronutrient intake and the prevalence of inadequate intake could contribute to the formulation of appropriate health intervention policies, in order to promote public health and reduce the socioeconomic burden.

Usual dietary intake is defined as the long-term average daily intake of a nutrient or food. Due to the within-individual variation, the average of short-term 24h recalls would lead to a biased estimate of the fraction of the population with usual intake above or below some standard. The National Cancer Institute (NCI) method was developed to address some of the measurement error issues inherent in the analysis of 24 h recall data [13,14]. The validity of the method has been proven, and it has been widely used in national nutrition surveys in Europe and the United States [15,16,17]. 

Most previous studies used the average of 24 h recall to describe the distribution of the micronutrient intake and the percentage of inadequate intake among the Chinese population [18,19], but there is a lack of research to estimate the usual micronutrient intake in the Chinese population. In this study, based on the data from China Nutrition and Health Surveillance 2015–2017 (CNHS), we evaluated the usual intakes of vitamins and minerals and the prevalence of inadequate intakes among Chinese adults aged 18 years and older by the NCI method. To our best knowledge, this is the first study to estimate the usual intake distribution and deficiency status of micronutrients for Chinese adults, and to weight the results so that they are nationally representative.

## 2. Materials and Methods

### 2.1. Study Subjects

All information on the subjects was obtained from CNHS 2015–2017. The multistage stratified whole-group random sampling method was used to draw participants aged 18 years and above, which allowed for a nationally representative sample. Subjects were selected from 298 surveillance sites in 31 provinces (autonomous regions and municipalities) for the nutrition and chronic disease survey. A detailed description of CNHS can be found in the previous publication [20]. Participants with no available information or abnormal energy intake (below 800 kcal/day or above 4200 kcal/day for males; below 600 kcal/day or above 3500 kcal/day for females) were excluded, and a total of 72,231 subjects aged 18 years and older were included in the current study.

The project was reviewed by the Ethics Review Committee of the Chinese Center for Disease Control and Prevention (No. 201519-B), and all subjects signed an informed consent form prior to the survey.

### 2.2. Data Collection

A standard questionnaire designed by the national project team was used to collect information by face-to-face questioning, including basic information, socio-demographic characteristics, physical activities, etc. The dietary data of the subjects were collected by the 24 h dietary recall method combined with the condiment weighing method for three consecutive days (two weekdays and one weekend). The body height and weight of the participants were measured using a TZG height meter (accuracy 0.1 cm) and a Paradigm HD-390 electronic weight scale (accuracy 0.1 kg). A detailed introduction to the data collection can be found in previous studies [21].

### 2.3. Micronutrients Analysis

The consumption of household cooking oil and condiments was assigned to individuals in proportion to the energy intake of household members and added to the consumption of various foods calculated by the 24 h dietary recall method, in order to obtain the daily food and condiment intake of the participants. According to Chinese Food Composition Table (2009 and 2018 edition) [22,23], the individual daily intake of energy; minerals (calcium, iron, zinc, copper, selenium, phosphorus, magnesium, manganese, sodium, potassium); and vitamins (vitamin A, vitamin E, vitamin C, thiamine, riboflavin, niacin) was calculated. Vitamin A intake was calculated as μg of retinol plus 1/6 μg of total carotene. Due to the limitations of the food composition tables, the usual intakes of vitamin B6, folic acid, vitamin B12, and vitamin D were not included in this analysis.

The estimated average requirement (EAR) cut-point method was applied to estimate the prevalence of inadequate micronutrient intake. Corresponding EAR values for each age–sex group were derived from the Chinese Dietary Reference Intakes (DRIs) 2013 [24]. For manganese, sodium, potassium, and vitamin E, the corresponding adequate intake (AI) was used to replace the EARs because these EARs for Chinese adults have not yet been established. The EARs and AIs of selected micronutrients for Chinese adults based on DRIs 2013 are shown in Supplementary Table S1.

### 2.4. Usual Intake Estimation

The National Cancer Institute (NCI) method was used to estimate the distribution of usual micronutrient intake and the prevalence of inadequacy based on specific age–sex DRIs. This method reduces measurement error by estimating and eliminating the within-individual variability in the diet. We estimated the usual intake at the group level by means of MIXTRAN and DISTRIB macros (version 2.1). The amount-model was conducted by adjusting for age (18~49 years, 50~64 years, 65~79 years, and 80~ years); sex (male and female); urban/rural; education level (illiteracy, primary or middle school, and high school or above); household income level (<20,000 CNY, 20,000~50,000 CNY, >50,000 CNY, and unclear); body mass index (BMI) (<18.5 kg/m^2^, 18.5~23.9 kg/m^2^, 24~27.9 kg/m^2^, and 28~ kg/m^2^); physical activities (low: <600 MET-min/week, medium: 600~3000 MET-min/week, high: >3000 MET-min/week); and sequence of 24 h recall (first recall, second recall, and third recall). The validity of the NCI method for estimating the usual dietary intake of Chinese adults has been demonstrated [17]. A more detailed description of the NCI method can be found at http://riskfactor.cancer.gov/diet/usualintakes/ (accessed on 2 October 2022).

### 2.5. Statistical Analysis

Mean, median, and quartiles were used to describe the distribution of usual intakes. To consider the complex stratified cluster sample design of the CNHS when estimating standard errors, we used the jackknife-n method to produce 93 replicate weights. The sampling weights were also used to obtain unbiased estimates. An unpaired t-test was used to test the distribution of usual intake and the prevalence of inadequate intake between different genders in the same age group. Replicate weights were created by Wesvar 5.1.19 software. The NCI method and other analyses were conducted by using SAS version 9.4 (SAS Institute Inc., Cary, NC, USA).

## 3. Results

### 3.1. Characteristics of Subjects

A total of 72,231 adults were included in the study. The number of females was slightly higher than males. The highest proportion of respondents was between 18 and 49 years old, while the lowest proportion was from 80 years old and above. There were more subjects from rural areas than urban areas (59.35% vs. 40.65%). The largest number of subjects graduated from primary or junior high school, accounting for 51.12%. The annual household income of the subjects was mainly in the range of 20,000~50,000 CNY. The percentage of subjects with normal BMI (18.5~23.9 kg/m^2^) was 46.76%. The proportion of participants with low, medium, and high levels of physical activity were 23.89%, 24.8%, and 51.31%, respectively. In addition, the distribution of characteristics was similar between males and females. The detailed information of the study population is presented in Table 1.

### 3.2. Prevalence of Inadequacy Micronutrient Intakes in Chinese Adults

**Figure 1:**
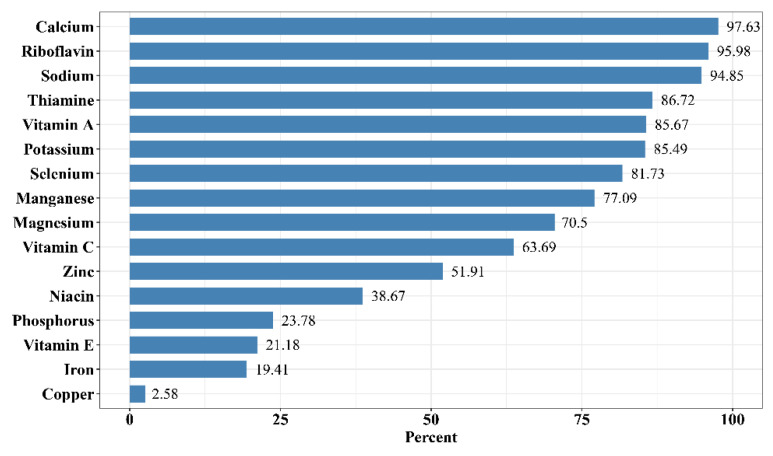
Percentage of Chinese adults with inadequate micronutrient intakes from diet based on EARs or AIs. The bar corresponding to sodium in the figure shows the percentage of Chinese adults with excess sodium intake over AI.

From the data analysis in the Figure 1 results, the micronutrient intakes of Chinese adults were seriously insufficient, with the prevalence of inadequate intake ranging from 2.58% to 97.63%. Calcium deficiency rate was the highest, followed by riboflavin, both with the percentage of inadequate intake exceeding 95%. The prevalence of inadequacy of thiamine, vitamin A, potassium, and selenium was very high, with the proportion of adults below the EARs or AIs at more than 80% (thiamine: 86.72%; vitamin A 85.67%; potassium: 85.49%; selenium: 81.73%). There were also potential deficiencies in manganese, magnesium, vitamin C, zinc, and niacin, as the percentage of inadequate intake of these micronutrients exceeded 30% (38.67% to 77.09%). It is noteworthy that the proportion of Chinese adults with a usual dietary sodium intake more than the AI reached 94.85%.

### 3.3. Distribution of Usual Mineral Intake, Prevalence of Inadequacy in Different Age–Sex Subgroups

The distribution of calcium and iron intakes and the level of their intake inadequacy based on EAR by age and gender is presented in Table 2. The highest calcium intake was in the 50~64 age group at 360.03 mg/day for males and 313.68 mg/day for females. The dietary calcium intake was significantly higher in males than in females in the same age group (*p* < 0.05). Most subjects (over 97%) showed inadequate calcium intake. The prevalence of calcium inadequacy among males in different age groups was lower than among females (*p* < 0.05). However, the prevalence of inadequate iron intake was significantly higher in females than in males in different age groups (*p* < 0.05), with a deficiency rate of 50.58% in females in the 18~49 years age group. In addition, the prevalence of inadequate calcium intake increased with age, as did the prevalence of inadequate iron intake in males.

As shown in Table 3, no significant gender difference was found in terms of usual zinc intake in the age groups of 65~79 years and 80 years and above. However, the prevalence of zinc inadequacy among males (range: 67.27~88.56%) in different age groups was significantly higher than among females (range: 30.59~60.52%; *p* < 0.05). Few participants had inadequate copper intake, and the rate of copper deficiency was significantly higher in females than in males (*p* < 0.05). It can be visualized that the rate of inadequate intake of zinc and copper increases with age.

Table 4 shows that the usual intake of selenium and phosphorus decreased with age and the percentage of their inadequate intake increased with age. The dietary selenium and phosphorus intake of the total population was 36.61 μg/day and 785.07 mg/day, respectively. The intakes of females in the same age group were significantly lower than those of males (*p* < 0.05). The percentage of inadequate selenium intake remained very high (from 72.44 to 96.18%) in all groups. The percentage of deficient intake of selenium and phosphorus was higher in females than in males (*p* < 0.05); in particular, the rate of inadequate phosphorus was twice as high in females as in males in the same age group.

As seen in Table 5, the highest average usual intake of both magnesium and manganese was in the age group of 50~64 years, which was slightly higher than in the age group of 18~49 years. The prevalence of inadequate magnesium intake was 70.5%, with a higher incidence in females than in males in the same age group (*p* < 0.05). As with selenium, the prevalence of inadequate intake was at a high level, ranging from 81.74 to 92.98% in females and 69.15 to 84.89% in males, with statistically significant differences by gender (*p* < 0.05).

The usual intake of potassium decreased with age, and the highest usual intake of sodium was in the age group of 50~64 years (Table 6). The intakes of males were a little higher than females in the same age group (*p* < 0.05). The mean and median intakes of sodium were much higher than AI for each respective age group. For example, the median of sodium in the 50~64 years group, 5253.12 mg/day in males and 4257.47 mg/day in females, was much higher than the AI of 1400 mg/day. In contrast, the mean and median intakes of potassium were less than the AI for each respective age group. As we age, the gaps between the recommended intake and the usual intake grow larger. The prevalence of inadequate potassium intake in the total population was more than 85%.

### 3.4. Distribution of Usual Vitamin Intake, Prevalence of Inadequacy in Different Age–Sex Subgroups

The distribution of usual intakes of vitamin A, vitamin E, vitamin C, thiamine, riboflavin, and niacin and the prevalence of their inadequate intake are shown in Table 7, Table 8 and Table 9. The average intakes of these vitamins in males were higher than in females in the same age group (*p* < 0.05). The average intakes of vitamin A, thiamine, riboflavin, and niacin were found to decrease with age, and the percentage of inadequate vitamins intake increased with age. However, the group with the lowest prevalence of inadequate intake of vitamin E and vitamin C was the 50~64 age group. Riboflavin, thiamine, and vitamin A deficiencies were the most severe, with the prevalence of inadequate intake exceeding 95%, 85%, and 83%, respectively. The prevalence of inadequate riboflavin and thiamine intake was not statistically significant differences between females and males in the same age group (*p* > 0.05). The percentage of subjects with dietary intakes of vitamin C, niacin, and vitamin E below the EAR were 63.69%, 38.67%, and 21.18%, respectively. Additionally, the percentage of females deficient in these three vitamins was higher than males in the same age group, except for niacin in the age group of 80 years and older. The prevalence of these micronutrient deficiencies was higher in females than in males, mainly due to low micronutrient intake caused by low food intake in females, while the EAR values of these micronutrients were consistent between females and males.

## 4. Discussion

Previous studies have mostly used the traditional average-value method to calculate the nutrients intakes for Chinese adults. Only two studies have estimated usual micronutrient intakes and the prevalence of inadequacy for Chinese adults. The first study estimated the usual nutrients intakes for adults aged 18 years and older using the Iowa State University (ISU) method, which is based on the CNHS 2002 data, and showed that high prevalence of the inadequate intake of calcium, zinc, selenium, magnesium, thiamine, and riboflavin in Chinese adults, with a deficiency rate of over 95% for calcium and over 80% for thiamine and riboflavin [25].The second study used the Multiple Source Method (MSM) to estimate the usual micronutrient intake status in people aged 60 years and more based on CNHS (2010–2012) data. This study showed that micronutrient intake deficiencies were severe among people over 60 years of age in China, with deficiencies in calcium, riboflavin, thiamine, selenium, and vitamin A of 98.2%, 94%, 87.7%, 81.1%, and 76.7%, respectively [26]. In the present study, we estimated the usual intake and adequacy of 16 micronutrients for Chinese adults by the NCI method based on the latest CNHS data. In addition, we explained the variance estimation of complex sampling by establishing repeated weights, and the findings were nationally representative.

In this study, we selected 16 micronutrients, including minerals and vitamins, for frequent evaluation. The results showed that Chinese adults were severely deficient in several essential micronutrients, such as calcium, selenium, potassium, vitamin A, thiamine, and riboflavin, with deficiencies in the intake of these nutrients exceeding 80%. These results are consistent with previous studies [25,26]. For most selected micronutrients, their intakes decreased with age, which is to be expected because older adults tend to consume less food and energy [27]. However, the age–sex EARs of certain nutrients may also decline with age, and the prevalence of inadequate intake increases with age, suggesting the rate of decrease increased in older adults. Among the minerals, the prevalence of inadequate calcium intake was the highest, and the usual dietary calcium intake was 327.98 mg/day, well below the EAR value. The main reason for this may be the low rate of consumption of calcium-rich dairy products by Chinese adults, such as milk, yogurt, and cheese. The milk consumption rate and the 95th percentile daily milk intake of Chinese adults aged 45 years and above in 2015 were 14.7% and 158.9 g/day, respectively [28].

Like calcium, usual dietary selenium, magnesium, manganese, and potassium was also low among Chinese adults, and the prevalence of inadequate intake was more than 70%. The prevalence of these minerals’ deficiencies was much higher in females than in males, especially among those aged 18~49 years. It is worth highlighting that, although the proportion of inadequate iron intake in the adults was 19.51%, the prevalence of inadequacy among females aged 18~49 years was more than 50%, which was much higher than the rest of the population. Women between the ages of 18 and 49 are of childbearing age and go through special physiological stages such as menstruation, pregnancy preparation, pregnancy, breastfeeding, and menopause, which increase their need for nutrients [29]. Compared with Americans, the prevalence of mineral inadequacies was much higher in Chinese females aged 18~49 years. For example, based on the National Health and Nutrition Examination Survey (NHANES) 2011–2016, the prevalence of inadequate dietary calcium, iron, magnesium, phosphorus, zinc, and selenium among females aged 15~30 years was 44.2%, 10,1%, 58.5%, <3%,17.6%, and <3%, while the inadequate intake of dietary calcium, iron, magnesium, phosphorus, zinc, and selenium among women aged 31–44 years was 43.7%,9.5%,43.7%, <3%, 14.5%, <3%, respectively [30]. In contrast to the above-mentioned higher rates of mineral deficiency in females than in males, the proportion of dietary zinc below EAR in males was significantly higher than in females, although males had a higher intake of zinc than females. The prevalence of zinc deficiency in males in the 18~49 and 50~64 age groups was 67.27% and 71.26%, respectively, which was twice as high as those in females in the same age group. Dietary zinc intake for American males aged 20 years and over was 13.2 mg/day, which was much higher than the 9.4 mg/day for American females aged 20 years and over, and the zinc intake of American adults was much higher than that of Chinses adults (11.2 mg/day vs. 8.34 mg/day) [31]. Males have a higher need for zinc, which leads to a higher rate of inadequate zinc intake, making it more important for them to pay attention to zinc supplementation. Dietary phytates are mainly found in plant foods such as legumes, refined grains, and tubers, which affect the bioavailability of minerals such as zinc and calcium [32,33]. Studies have shown that the dietary phytate intake of people in China is higher than those in Western developed countries [34], so Chinese people with an inadequate intake of minerals such as calcium, zinc, and iron should adjust their diet patterns avoid further deficiency.

The results of the usual sodium and potassium intake estimates showed that Chinese adults had a serious excess of sodium intake and a serious deficiency of potassium intake, which was more severe in the older age group. More than 25% of adults with usual sodium intake in all age–sex groups exceed the World Health Organization (WHO) recommendation (2000 mg/day) [35], while less than 15% of adults with usual potassium intake meet AI level. In 2017, China had the highest cardiovascular disease and cancer mortality caused by poor dietary habits among countries with a large population, and a high intake of sodium was the leading dietary risk for deaths and disability-adjusted life years (DALYs) [2]. Additionally, a strong body of evidence documents the efficacy of sodium reduction and potassium supplementation in lowering blood pressure [36]. Meta-analyses of cohort studies have consistently noted a strong significant inverse relationship between dietary potassium intake and CVD [37]. A randomized controlled trial showed a greater effect of potassium supplementation on lowering blood pressure in adults with higher sodium–potassium ratios [38], and another prospective analysis showed similar results, in that sodium–potassium ratios correlated more strongly with CVD than sodium or potassium alone [39]. Therefore, Chinese adults should simultaneously control their sodium intake and supplement their potassium deficiency, for example, increase their intake of vegetables, fruits, and legumes, and reduce the intake of sodium salt and processed meat products.

The prevalence of riboflavin inadequacy was the highest among the vitamins in this study (95.98%), with the average usual dietary riboflavin intake of merely 0.62 mg/day in Chinese adults. Like riboflavin, the usual intake of Chinese adults was very low in thiamine and vitamin A, with more than 85% below the EAR. There was no statistically significant difference in the prevalence of inadequate intake of thiamine and riboflavin between the genders in the same age group. The percentage of inadequate vitamin E and vitamin C intake was higher in females than in males, while males had a higher proportion of vitamin A deficiency in the same age groups. A previous study showed that in 2015, the prevalence of vitamin A deficiency among 15 provinces in China was 66.39% for males aged 18–35 years and 63.39% for females aged 18–35 years [40], which was much lower than the 86.51% for males and 83.33% for females in this study. The main reason for this difference may be due to the different methods used to calculate vitamin A intake. The amount of vitamin A in different foods varies widely, which results in much greater intra-individual variation in vitamin A than in other nutrients. Therefore, the vitamin A intake estimated by the traditional average method was used to calculate the proportion of vitamin A intake below the EAR, which led to an underestimation of the true prevalence of inadequate intake [41,42]. Compared with data from NHANES 2005–2016, the prevalence of inadequate usual vitamin intake from food alone was much higher in Chinese adults (≥18 years) than in American adults (>19 years), except for vitamin E (21.18% of Chinese adults vs. 83.7% of American adults), such as vitamin A (85.67% vs. 45.21%); vitamin C (63.69% vs. 45.92%); thiamine (86.72% vs. 6.57%); riboflavin (95.98% vs. 2.86%); and niacin (38.67% vs, 1.43%) [43]. However, it should be noted that due to the limitations of the food composition table used, the micronutrient content of fortified foods was not included in the calculation of the dietary micronutrients in this study. Since some commonly consumed foods are enriched or fortified in the United States, the gap between the results of this study and adult micronutrient deficiency rates in the United States may be overestimated.

Over the past 20 years, the dietary patterns in China have changed; the consumption of edible oils, meat, refined grains, and sugars in the Chinese population has increased rapidly; while the consumption of whole grains, fruits, and vegetables has continually declined [21,44,45]. In 2015, the consumption of vegetables and fruits among Chinese elderly consumers over 60 years old was 300 g/day and 50 g/day, respectively, and only half of the consumers consumed up to the recommended value of Chinese dietary guidelines, while the percentage of consumers with an insufficient fruit intake was more than 85% [46]. The results from CNHS 2010–2012 showed that the consumption of edible oil among Chinese residents aged 2 years and above is 41.8 g, of which 37.3 g are vegetable oils and 4.8 g are animal oils, indicating a high consumption of edible oil by Chinese residents [47]. This is the reason why Chinese adults were severely deficient in B-vitamins and vitamin C, while vitamin E intake was adequate. A major cause of micronutrient deficiency is the low intake of micronutrient-rich foods. For instance, calcium deficiency was severe among the Chinese population, since dairy products intake was so low. Data show that the main food source of dietary calcium was vegetables and cereals, while the proportion of milk and legumes was less than 20%. In general, our recommendation to improve calcium deficiency is to advocate increasing the intake of calcium-rich foods. However, due to people’s inherent eating habits and preferences, these suggestions are difficult to adopt. Therefore, fortified calcium in foods that people commonly consume may be a more effective way to address calcium deficiency [48].

One of the strengths of this study was the use of the most recent nationally representative nutrition survey data. Secondly, we calculated usual micronutrients intake using the NCI model adjusted for covariates that might affect intake, which reduced the measurement error of the 24 h dietary recall [49]. Finally, we created 93 repeated weights for explaining the variance estimation of the complex sampling design. However, the present study still had several limitations. Firstly, we estimated the usual micronutrients intake from food alone and did not consider dietary supplements, which may overestimate the prevalence of inadequate micronutrient intake. Since the consumption rate of dietary supplements among Chinese adults was low, the overestimation of the extent of inadequate intake should be limited. In addition, the usual intakes of vitamin B6, folic acid, vitamin B12, and vitamin D, which are also important for human health, were not estimated due to the limitations of the food composition tables.

## 5. Conclusions

In conclusion, Chinese adults aged 18 years and above have an insufficient intake of multiple micronutrients, except for sodium, copper, iron (only for males), vitamin E, and phosphorus. Compared to the reference values of DRIs 2013, a substantial proportion of Chinese adults had an inadequate intake of calcium, riboflavin, thiamine, vitamin A, potassium, and selenium. The usual dietary intake of manganese, magnesium, vitamin C, zinc, and niacin among Chinese adults was potential deficiency, while the usual sodium intake was serious excess. The prevalence of inadequate micronutrient intake increased with age, and the females were more vulnerable to micronutrients deficiency.

Therefore, there is an urgent need for effective strategies and measures to improve the micronutrient deficiencies in Chinese adults. In addition, we encourage other countries to use the same method of reducing measurement error to estimate the prevalence of nutrient intakes in their populations and to explore any solutions that could change the status of micronutrient deficiencies. 

## Figures and Tables

**Table 1:** Characteristics of subjects aged 18 years and older by gender in CNHS 2015–2017.

Characteristics	Total (%)	Male (%)	Female (%)
Total	72,231 (100)	34,011 (47.09)	38,220 (52.91)
Age (year)			
18~49	29,900 (41.39)	13,414 (39.44)	16,486 (43.13)
50~64	27,509 (38.08)	12,909 (37.96)	14,600 (38.2)
65~79	13,412 (18.57)	6951 (20.44)	6461 (16.9)
80~	1410 (1.95)	737 (2.17)	673 (1.76)
Residence			
Urban	29,359 (40.65)	13,514 (39.73)	15,845 (41.46)
Rural	42,872 (59.35)	20,497 (60.27)	22,375 (58.54)
Education level			
Illiteracy	20,607 (28.53)	6539 (19.23)	14,068 (36.81)
Primary or middle school	36,925 (51.12)	19,469 (57.24)	17,456 (45.67)
High school or above	14,699 (20.35)	8003 (23.53)	6696 (17.52)
Household income level (CNY)			
<20,000	15,646 (21.66)	7576 (22.28)	8070 (21.11)
20,000~50,000	24,367 (33.73)	11,418 (33.57)	12,949 (33.88)
>50,000	20,437 (28.29)	9587 (28.19)	10,850 (28.39)
Unclear	11,781 (16.31)	5430 (15.97)	6351 (16.62)
BMI (kg/m^2^)			
<18.5	2775 (3.84)	1220 (3.59)	1555 (4.07)
18.5~23.9	33,778 (46.76)	16,017 (47.09)	17,761 (46.47)
24~27.9	25,384 (35.14)	12,224 (35.94)	13,160 (34.43)
28~	10,294 (14.25)	4550 (13.38)	5744 (15.03)
Physical activity			
Low	17,257 (23.89)	9071 (26.67)	8186 (21.42)
Medium	17914 (24.8)	7788 (22.9)	10,126 (26.49)
High	37,060 (51.31)	17,152 (50.43)	19,908 (52.09)

Abbreviation: BMI—Body Mass Index; CNY—Chinese Yuan.

**Table 2:** Usual intake and prevalence of intake inadequacy for calcium and iron among Chinese adults.

Age Group	Calcium (mg/Day)					Iron (mg/Day)				
	Mean * (SE)	P25 * (SE)	Median * (SE)	P75 * (SE)	%Below EAR * (SE)	Mean * (SE)	P25 * (SE)	Median * (SE)	P75 * (SE)	%Below EAR * (SE)
Males										
18~49	350.18 (4.59)	244.4 (3.53)	322.91 (4.16)	425.07 (5.83)	95.79 (0.41)	18.91 (0.19)	14.33 (0.17)	17.93 (0.19)	22.37 (0.23)	1.84 (0.24)
50~64	360.03 (4.66)	252.42 (3.78)	331.76 (4.44)	436.48 (5.83)	98.53 (0.16)	18.72 (0.19)	14.22 (0.15)	17.74 (0.18)	22.15 (0.23)	1.99 (0.22)
65~79	333.98 (5.09)	233.15 (4.23)	307.46 (4.9)	404.63 (6.16)	99.04 (0.12)	16.54 (0.16)	12.54 (0.12)	15.67 (0.15)	19.57 (0.19)	4.76 (0.37)
80~	304.94 (8.57)	212.1 (6.16)	280.25 (7.96)	370.91 (10.85)	99.49 (0.13)	14.7 (0.32)	11.12 (0.25)	13.91 (0.31)	17.43 (0.38)	9.52 (1.22)
Females										
18~49	306.65 (4.58)	214.09 (2.83)	282.51 (3.93)	372.17 (6)	98.01 (0.27)	15.75 (0.14)	11.94 (0.12)	14.93 (0.14)	18.65 (0.18)	50.58 (1.1)
50~64	313.68 (4.95)	219.09 (3.42)	288.96 (4.44)	380.52 (6.36)	99.39 (0.1)	15.62 (0.15)	11.84 (0.11)	14.81 (0.14)	18.49 (0.19)	6.69 (0.43)
65~79	290.26 (5.33)	202.36 (3.81)	266.99 (4.84)	352.26 (6.66)	99.65 (0.08)	13.75 (0.13)	10.4 (0.09)	13.02 (0.11)	16.3 (0.17)	13.21 (0.6)
80~	262.73 (7.58)	182.86 (5.46)	241.78 (7.05)	317.86 (9.94)	99.8 (0.06)	12.12 (0.26)	9.14 (0.2)	11.48 (0.24)	14.35 (0.3)	23.55 (2.45)
Total	327.98 (3.94)	227.55 (2.83)	301.43 (3.51)	398.63 (5.03)	97.63 (0.25)	17.02 (0.14)	12.7 (0.11)	16.05 (0.13)	20.27 (0.17)	19.41 (0.51)

*—Significant at *p* < 0.05 between age–sex groups.

**Table 3:** Usual intake and the prevalence of intake inadequacy for zinc and copper among Chinese adults.

Age Group	Zinc (mg/Day)					Copper (mg/Day)				
	Mean (SE)	P25 (SE)	Median (SE)	P75 (SE)	%Below EAR * (SE)	Mean * (SE)	P25 * (SE)	Median * (SE)	P75 * (SE)	%Below EAR * (SE)
Males										
18~49	9.46 (0.29) *	7.11 (0.49) *	8.94 (0.38) *	11.23 (0.27) *	67.27 (2.97)	1.49 (0.02)	1.09 (0.02)	1.4 (0.02)	1.79 (0.03)	1.16 (0.16)
50~64	9.11 (0.76) *	6.86 (0.87)	8.61 (0.83)	10.8 (0.75) *	71.26 (7.24)	1.47 (0.02)	1.07 (0.01)	1.38 (0.02)	1.77 (0.02)	1.26 (0.15)
65~79	8.09 (0.58)	6.08 (0.69)	7.64 (0.65)	9.59 (0.56)	81.93 (3.66)	1.35 (0.02)	0.98 (0.01)	1.26 (0.02)	1.62 (0.02)	2.33 (0.27)
80~	7.32 (0.83)	5.5 (0.84)	6.91 (0.88)	8.71 (0.91)	88.56 (4.14)	1.22 (0.02)	0.89 (0.02)	1.14 (0.02)	1.47 (0.03)	4.21 (0.58)
Females										
18~49	7.67 (0.46)	5.77 (0.12)	7.25 (0.32)	9.1 (0.67)	30.59 (2.63)	1.27 (0.02)	0.92 (0.01)	1.19 (0.01)	1.52 (0.02)	3.34 (0.29)
50~64	7.4 (0.13)	5.56 (0.19)	7 (0.09)	8.79 (0.27)	34.32 (2.43)	1.25 (0.01)	0.91 (0.01)	1.17 (0.01)	1.5 (0.02)	3.68 (0.31)
65~79	6.56 (0.25)	4.92 (0.09)	6.19 (0.16)	7.79 (0.41)	48.33 (2.69)	1.14 (0.01)	0.83 (0.01)	1.07 (0.01)	1.37 (0.02)	6.09 (0.48)
80~	5.9 (0.24)	4.42 (0.25)	5.57 (0.25)	6.98 (0.3)	60.52 (4.67)	1.03 (0.02)	0.74 (0.02)	0.96 (0.02)	1.23 (0.02)	10.36 (0.92)
Total	8.34 (0.12)	6.14 (0.23)	7.83 (0.15)	9.98 (0.17)	51.91 (1.71)	1.36 (0.02)	0.98 (0.01)	1.27 (0.01)	1.64 (0.02)	2.58 (0.22)

*—Significant at *p* < 0.05 between age–sex groups.

**Table 4:** Usual intake and prevalence of intake inadequacy for selenium and phosphorus among Chinese adults.

Age Group	Selenium (μg/Day)					Phosphorus (mg/Day)				
	Mean * (SE)	P25 * (SE)	Median * (SE)	P75 * (SE)	%Below EAR * (SE)	Mean * (SE)	P25 * (SE)	Median * (SE)	P75 * (SE)	%Below EAR * (SE)
Males										
18~49	42.25 (0.82)	28.76 (0.54)	38.68 (0.7)	51.73 (1.05)	72.44 (1.56)	877.78 (7.86)	690.91 (7.31)	844.63 (7.65)	1026.77 (9.12)	12.54 (0.79)
50~64	38.87 (0.62)	26.48 (0.52)	35.51 (0.62)	47.57 (0.78)	78.57 (1.12)	857.12 (7.79)	675.91 (7.29)	823.86 (7.94)	1002.82 (8.88)	14.22 (0.87)
65~79	34.24 (0.49)	23.16 (0.45)	31.2 (0.52)	41.87 (0.6)	86.05 (0.69)	771.61 (7.67)	605.72 (6.86)	741.08 (7.63)	903.44 (8.87)	22.29 (1.13)
80~	31.96 (0.73)	21.54 (0.57)	29.1 (0.74)	39.31 (0.92)	89.16 (0.91)	706.12 (11.36)	552.23 (9.35)	676.6 (11.05)	828.83 (13.95)	26.32 (1.68)
Females										
18~49	34.16 (0.47)	23.17 (0.27)	31.2 (0.38)	41.84 (0.61)	86.2 (0.88)	725.46 (5.64)	569.04 (4.71)	696.89 (5.52)	850.57 (7.08)	30.92 (0.92)
50~64	31.04 (0.32)	20.98 (0.28)	28.31 (0.33)	38.03 (0.41)	90.39 (0.48)	706.68 (5.62)	553.72 (5.01)	678.71 (5.61)	828.7 (6.87)	34.09 (1.07)
65~79	27.28 (0.32)	18.36 (0.28)	24.81 (0.33)	33.46 (0.41)	94.49 (0.38)	633.38 (5.84)	494.35 (4.84)	606.9 (5.82)	743.86 (7.13)	46.23 (1.26)
80~	25.22 (0.59)	16.9 (0.46)	22.97 (0.54)	30.74 (0.7)	96.18 (0.56)	574.89 (9.44)	446.12 (7.94)	551.01 (9.07)	674.89 (11.46)	52.34 (2.4)
Total	36.61 (0.5)	24.32 (0.31)	33.18 (0.42)	45.08 (0.65)	81.73 (0.93)	785.07 (5.64)	605.47 (4.86)	750.82 (5.58)	927.3 (6.88)	23.78 (0.77)

*—Significant at *p* < 0.05 between age–sex groups.

**Table 5:** Usual intake and prevalence of intake inadequacy for magnesium and manganese among Chinese adults.

Age Group	Magnesium (mg/Day)					Manganese (mg/Day)				
	Mean * (SE)	P25 * (SE)	Median * (SE)	P75 * (SE)	%Below EAR * (SE)	Mean * (SE)	P25 * (SE)	Median * (SE)	P75 * (SE)	%Below AI * (SE)
Males										
18~49	268.3 (2.16)	206.28 (2.06)	255.48 (2.15)	315.84 (2.5)	61.36 (1.02)	3.93 (0.04)	2.69 (0.04)	3.59 (0.04)	4.78 (0.05)	70.2 (0.85)
50~64	275.04 (2.83)	211.93 (2.38)	261.86 (2.77)	323.83 (3.35)	58.45 (1.29)	3.98 (0.04)	2.73 (0.03)	3.64 (0.04)	4.84 (0.06)	69.15 (0.9)
65~79	249.9 (3.14)	192.05 (2.65)	237.7 (3.06)	294.16 (3.64)	65.66 (1.45)	3.53 (0.05)	2.42 (0.04)	3.23 (0.04)	4.3 (0.06)	78.28 (0.9)
80~	222.39 (3.84)	170.79 (3.05)	211.4 (3.59)	262.33 (4.61)	74.13 (1.68)	3.16 (0.08)	2.16 (0.06)	2.88 (0.08)	3.85 (0.09)	84.89 (1.41)
Females										
18~49	226.3 (1.71)	173.86 (1.47)	215.35 (1.62)	266.65 (2.16)	79.68 (0.74)	3.29 (0.03)	2.26 (0.02)	3.01 (0.03)	4.02 (0.04)	82.68 (0.71)
50~64	231.55 (2.54)	178 (2.04)	220.5 (2.39)	272.67 (3.11)	77.61 (1.09)	3.35 (0.04)	2.29 (0.02)	3.06 (0.03)	4.08 (0.06)	81.74 (0.91)
65~79	209.54 (2.6)	160.78 (2.1)	199.19 (2.48)	247.05 (3)	83.01 (1.04)	2.96 (0.04)	2.02 (0.03)	2.7 (0.04)	3.61 (0.05)	88.4 (0.77)
80~	185.26 (3.43)	141.92 (2.69)	176.07 (3.24)	218.53 (4.15)	88.81 (1.13)	2.62 (0.06)	1.78 (0.05)	2.39 (0.06)	3.19 (0.07)	92.98 (0.68)
Total	246.22 (1.78)	186.78 (1.57)	233.46 (1.72)	291.54 (2.13)	70.5 (0.78)	3.57 (0.03)	2.42 (0.02)	3.25 (0.03)	4.37 (0.04)	77.09 (0.65)

*—Significant at *p* < 0.05 between age–sex groups.

**Table 6:** Usual intake and prevalence of intake inadequacy for sodium and potassium among Chinese adults.

Age Group	Sodium (mg/Day)					Potassium (mg/Day)				
	Mean * (SE)	P25 * (SE)	Median * (SE)	P75 * (SE)	%Below AI * (SE)	Mean * (SE)	P25 * (SE)	Median * (SE)	P75 * (SE)	%Below AI * (SE)
Males										
18~49	5721.7 (63.5)	3503.7 (55.9)	5209.6 (63.9)	7368.9 (78.2)	3.22 (0.3)	1615.7 (17.4)	1225.8 (16.4)	1538.6 (17.4)	1918.1 (20)	78.9 (0.92)
50~64	5765.4 (62.5)	3546.7 (49.5)	5253.1 (55.7)	7428.1 (77.9)	2.56 (0.25)	1585.1 (15.1)	1205.9 (14.8)	1507.9 (15.7)	1880.8 (17.3)	80.65 (0.76)
65~79	5216.4 (80.5)	3129.6 (58.3)	4716.7 (70.5)	6749 (106.9)	3.99 (0.31)	1444.3 (13.4)	1092.3 (13.5)	1371.9 (13.8)	1714.7 (14.9)	87.31 (0.56)
80~	4576.2 (126.3)	2651.4 (93.7)	4091.7 (125.5)	5991.6 (152.8)	5.63 (0.75)	1272.1 (26.8)	956 (21.1)	1205.6 (25.6)	1516 (32.7)	93.43 (0.86)
Females										
18~49	4649.4 (46.1)	2714.2 (39.8)	4171 (46.8)	6067.7 (61.1)	7.26 (0.46)	1385.5 (14.4)	1047.8 (12.1)	1316.8 (13.9)	1647.8 (17.7)	89.8 (0.67)
50~64	4736.4 (44.2)	2780.1 (37.1)	4257.5 (40.7)	6164.3 (59)	5.81 (0.43)	1353.6 (14)	1022.3 (12.2)	1286.1 (13.8)	1609.6 (17)	90.93 (0.59)
65~79	4255.1 (59.9)	2425.9 (40.8)	3783.9 (55.7)	5582.7 (81.2)	8.3 (0.53)	1227.4 (13)	923.4 (11.2)	1163.5 (12.9)	1462.4 (15.8)	94.82 (0.39)
80~	3659.8 (97.8)	2001.6 (97.3)	3217.6 (91.5)	4825.6 (129.5)	11.29 (1.12)	1070.6 (23.1)	801.1 (18.4)	1013.1 (22.2)	1272.7 (28.2)	97.91 (0.48)
Total	5139.6 (45.9)	3029.2 (39.4)	4624.3 (43.2)	6687.8 (60.7)	5.15 (0.36)	1472.4 (12.8)	1101.9 (11.7)	1395.3 (12.9)	1758.1 (15.2)	85.49 (0.61)

*—Significant at *p* < 0.05 between age–sex groups.

**Table 7:** Usual intake and prevalence of intake inadequacy for vitamin A and vitamin E among Chinese adults.

Age Group	Vitamin A (μgRAE/Day)					Vitamin E (mg/Day)				
	Mean * (SE)	P25 * (SE)	Median * (SE)	P75 * (SE)	%Below EAR * (SE)	Mean * (SE)	P25 * (SE)	Median * (SE)	P75 * (SE)	%Below AI * (SE)
Males										
18~49	327.07 (7.89)	164.18 (4.23)	271.63 (6.34)	428.18 (10.5)	86.51 (0.926)	29.72 (0.32)	16.8 (0.23)	26.47 (0.31)	39.05 (0.42)	17.98 (0.48)
50~64	313.59 (7.66)	157.58 (4.63)	259.51 (6.48)	409.96 (9.95)	87.94 (0.81)	30.53 (0.31)	17.44 (0.24)	27.24 (0.3)	40.08 (0.4)	16.67 (0.51)
65~79	299.97 (7.3)	148.13 (4.41)	246.45 (6.21)	391.61 (9.32)	89.13 (0.741)	27.28 (0.39)	15.02 (0.28)	24.06 (0.38)	36.01 (0.52)	22.2 (0.71)
80~	281.46 (17.43)	137.01 (10)	229.92 (15.12)	370.31 (23.31)	90.7 (1.582)	25.58 (0.82)	13.81 (0.57)	22.42 (0.81)	33.92 (1.04)	25.53 (1.56)
Females										
18~49	303.89 (7.41)	150.42 (3.85)	250.72 (5.99)	397.85 (9.7)	83.33 (1.04)	26.48 (0.32)	14.48 (0.2)	23.33 (0.31)	35.04 (0.44)	23.62 (0.54)
50~64	288.74 (7.08)	141.8 (4.08)	237.12 (5.93)	378.1 (9.15)	85.16 (0.908)	26.99 (0.24)	14.84 (0.19)	23.84 (0.25)	35.68 (0.34)	22.67 (0.5)
65~79	274.95 (7.22)	133.72 (4.21)	224.56 (6.12)	360.44 (9.52)	86.8 (0.835)	23.95 (0.36)	12.65 (0.22)	20.84 (0.35)	31.94 (0.5)	29.23 (0.8)
80~	254.67 (15.76)	122.45 (8.73)	207.38 (14.23)	332.75 (21.28)	88.94 (1.658)	22.21 (0.71)	11.4 (0.48)	19.16 (0.65)	29.61 (0.92)	33.41 (1.86)
Total	308.81 (6.51)	153.03 (3.5)	254.68 (5.13)	404.31 (8.53)	85.67 (0.835)	27.93 (0.25)	15.43 (0.17)	24.69 (0.25)	36.89 (0.35)	21.18 (0.4)

*—Significant at *p* < 0.05 between age–sex groups.

**Table 8:** Usual intake and prevalence of intake inadequacy for vitamin C and thiamine among Chinese adults.

Age Group	Vitamin C (mg/Day)					Thiamine (mg/Day)				
	Mean * (SE)	P25 * (SE)	Median * (SE)	P75 * (SE)	%Below EAR * (SE)	Mean * (SE)	P25 * (SE)	Median * (SE)	P75 * (SE)	%Below EAR (SE)
Males										
18~49	78.69 (1.36)	49.13 (1.12)	71.97 (1.3)	100.8 (1.67)	62.65 (1.29)	0.88 (0.01)	0.65 (0.01)	0.83 (0.01)	1.06 (0.01)	85.27 (0.84)
50~64	83.08 (1.4)	52.59 (1.11)	76.18 (1.32)	106.06 (1.73)	58.54 (1.3)	0.85 (0.01)	0.63 (0.01)	0.81 (0.01)	1.03 (0.01)	87.17 (0.74)
65~79	78.11 (1.59)	48.77 (1.29)	71.33 (1.53)	99.97 (1.89)	63.38 (1.52)	0.78 (0.01)	0.57 (0.01)	0.73 (0.01)	0.93 (0.01)	91.86 (0.54)
80~	67.42 (2.89)	40.8 (2.23)	60.83 (2.75)	87.1 (3.58)	73.31 (2.86)	0.69 (0.02)	0.51 (0.02)	0.65 (0.02)	0.84 (0.02)	95.65 (0.66)
Females										
18~49	75.2 (1.41)	46.63 (1)	68.54 (1.3)	96.56 (1.79)	66.09 (1.36)	0.72 (0.01)	0.53 (0.01)	0.68 (0.01)	0.87 (0.01)	85.68 (0.66)
50~64	79.66 (1.58)	49.94 (1.13)	72.91 (1.46)	102 (1.98)	61.78 (1.52)	0.7 (0.01)	0.51 (0.01)	0.66 (0.01)	0.84 (0.01)	87.53 (0.62)
65~79	74.48 (1.8)	45.91 (1.33)	67.71 (1.71)	95.72 (2.23)	66.77 (1.71)	0.63 (0.01)	0.46 (0.01)	0.6 (0.01)	0.77 (0.01)	92.36 (0.46)
80~	63.7 (2.81)	37.93 (2.29)	57.25 (2.81)	82.15 (3.57)	77.08 (2.51)	0.56 (0.02)	0.41 (0.01)	0.53 (0.01)	0.68 (0.02)	96.03 (0.68)
Total	77.67 (1.27)	48.33 (0.97)	70.91 (1.19)	99.61 (1.6)	63.69 (1.21)	0.78 (0.01)	0.57 (0.01)	0.74 (0.01)	0.95 (0.01)	86.72 (0.62)

*—Significant at *p* < 0.05 between age–sex groups.

**Table 9:** Usual intake and prevalence of intake inadequacy for riboflavin and niacin among Chinese adults.

Age Group	Riboflavin (mg/Day)					Niacin (mg/Day)				
	Mean * (SE)	P25 * (SE)	Median * (SE)	P75 * (SE)	%Below EAR (SE)	Mean * (SE)	P25 * (SE)	Median * (SE)	P75 * (SE)	%Below EAR * (SE)
Males										
18~49	0.69 (0.01)	0.5 (0.01)	0.64 (0.01)	0.82 (0.01)	95.63 (0.41)	15.02 (0.24)	10.67 (0.18)	14.07 (0.22)	18.31 (0.3)	34.75 (1.47)
50~64	0.66 (0.01)	0.48 (0.01)	0.62 (0.01)	0.79 (0.01)	96.62 (0.24)	14.27 (0.22)	10.14 (0.17)	13.35 (0.21)	17.39 (0.28)	39.57 (1.52)
65~79	0.6 (0.01)	0.43 (0.01)	0.56 (0.01)	0.71 (0.01)	98.13 (0.17)	12.56 (0.18)	8.83 (0.13)	11.71 (0.17)	15.34 (0.24)	43.96 (1.36)
80~	0.54 (0.02)	0.39 (0.01)	0.5 (0.01)	0.65 (0.02)	99.01 (0.2)	11.33 (0.38)	7.91 (0.28)	10.54 (0.36)	13.92 (0.47)	54.18 (3.29)
Females										
18~49	0.58 (0.01)	0.42 (0.01)	0.54 (0.01)	0.7 (0.01)	95.2 (0.41)	12.12 (0.14)	8.5 (0.11)	11.3 (0.13)	14.84 (0.18)	38.46 (1.03)
50~64	0.55 (0.01)	0.4 (0)	0.52 (0.01)	0.66 (0.01)	96.37 (0.28)	11.56 (0.14)	8.08 (0.1)	10.77 (0.13)	14.17 (0.18)	42.97 (1.11)
65~79	0.5 (0.01)	0.36 (0)	0.46 (0.01)	0.6 (0.01)	98.11 (0.19)	10.1 (0.13)	6.99 (0.08)	9.37 (0.12)	12.42 (0.18)	46.26 (1.09)
80~	0.45 (0.01)	0.33 (0.01)	0.42 (0.01)	0.54 (0.02)	99.01 (0.18)	9.01 (0.32)	6.19 (0.25)	8.35 (0.31)	11.05 (0.4)	45.94 (3.47)
Total	0.62 (0.01)	0.44 (0.01)	0.57 (0.01)	0.74 (0.01)	95.98 (0.31)	13.15 (0.17)	9.09 (0.11)	12.21 (0.15)	16.19 (0.22)	38.67 (1.09)

*—Significant at *p* < 0.05 between age–sex groups.

## Data Availability

The data presented in this study are not public.

## Supplementary Materials

The following supporting information can be downloaded at: https://www.mdpi.com/article/10.3390/nu14224714/s1, Table S1: EARs or AIs for selected micronutrients for Chinese adults based on DRIs (2013).
