# Supplementary material for: Usual Intake of Micronutrients and Prevalence of Inadequate Intake among Chinese Adults: Data from CNHS 2015–2017
Source: Nutrients. 2022 Nov 8;14(22):4714. doi: 10.3390/nu14224714 (PMC9696081; doi:10.3390/nu14224714)
Supplement: Supplementary file 1 [file nutrients-14-04714-s001.zip › nutrients-1988211-supplementary.pdf]

Table S1. EARs or AIs for selected micronutrients for Chinese adults based on DRIs (2013).

Age group	EAR											AI			
	Calcium (mg/d)	Iron (mg/d)	Copper (mg/d)	Selenium (μg/d)	Phosphorus (mg/d)	Magnesium (mg/d)	Vitamin A (μgRAE/d)	Vitamin C (mg/d)	Thiamine (mg/d)	Riboflavin (mg/d)	Niacin (mg/d)	Manganese (mg/d)	Sodium (mg/d)	Potassium (mg/d)	Vitamin E (mg/d)
Male															
18~49	650	9	0.6	50	600	280	560	85	1.2	1.2	12	4.5	1500	2000	14
50~64	800	9	0.6	50	600	280	560	85	1.2	1.2	12	4.5	1400	2000	14
65~79	800	9	0.6	50	590	270	560	85	1.2	1.2	11	4.5	1400	2000	14
80~	800	9	0.6	50	560	260	560	85	1.2	1.2	11	4.5	1300	2000	14
Female															
18~49	650	15	0.6	50	600	280	480	85	1.0	1.0	10	4.5	1500	2000	14
50~64	800	9	0.6	50	600	280	480	85	1.0	1.0	10	4.5	1400	2000	14
65~79	800	9	0.6	50	590	270	480	85	1.0	1.0	9	4.5	1400	2000	14
80~	800	9	0.6	50	560	260	480	85	1.0	1.0	8	4.5	1300	2000	14

DRIs— Chinese Dietary Reference Intakes; EAR— Estimated Average Requirement; AI— Adequate Intake.
